# Effects of a Combined Dietary and Physical Activity Intervention on Bone Density, Lean Mass and Fat Mass in Adults: The GOTO Trial

**DOI:** 10.1002/jcsm.70226

**Published:** 2026-03-08

**Authors:** F. A. Bogaards, Inge Groenendijk, Thies Gehrmann, Marian Beekman, Nico Lakenberg, H. Eka D. Suchiman, Lisette C. P. G. M. de Groot, Marcel J. T. Reinders, P. Eline Slagboom

**Affiliations:** ^1^ Molecular Epidemiology Leiden University Medical Center Leiden the Netherlands; ^2^ Leiden Computational Biology Center Leiden the Netherlands; ^3^ Division of Human Nutrition and Health Wageningen University & Research Wageningen the Netherlands; ^4^ Department of Bioscience Engineering, Lab of Applied Microbiology and Biotechnology University of Antwerp Antwerp Belgium; ^5^ Delft Bioinformatics Lab Delft University of Technology Delft the Netherlands; ^6^ Max Planck Institute for Biology of Aging Cologne Germany

**Keywords:** bone mineral density, caloric restriction, composite health scores, fat mass, lean mass, lifestyle intervention, physical activity

## Abstract

**Background:**

Nutritional weight‐loss interventions are known to reduce bone mineral density (BMD), which can be prevented by adding (resistance) exercise training. However, this combined effect is not well studied in non‐obese adults. In addition, the association between biomarkers and metabolite‐based composite health markers with changes in BMD in such an intervention has not been studied as thoroughly.

**Objective:**

The aims of the current study were to investigate the effect of a combined nutritional and activity lifestyle intervention on lumbar spine and total body BMD in healthy middle‐aged to older adults, and to relate these effects to a selection of immune‐metabolic biomarkers, muscle mass and fat mass measurements, and two composite metabolite‐based health scores.

**Methods:**

In this ancillary study of the single‐arm Growing Old TOgether (GOTO) trial (trial registration number GOTNL3301 [https://onderzoekmetmensen.nl/nl/trial/27183], NL‐OMON27183), 134 participants (mean age 62.9 years, 49% female) undertook a 13‐week lifestyle modification, incorporating 12.5% caloric restriction and 12.5% increase in physical activity. The impact on lumbar spine and total body BMD was evaluated using dual‐energy X‐ray absorptiometry (DEXA). The intervention effect on BMD was related to changes in immune‐metabolic biomarkers and two metabolite‐based immune‐metabolic health scores.

**Results:**

The trial significantly reduced bodyweight with 3.3 and 3.4 kg, consisting of 1.4 and 1.1 kg lean mass, in males (fdr < 0.001) and females (fdr < 0.001), respectively. Lean mass reduced by 1.4 kg in males (fdr < 0.001) and 1.1 kg in females (fdr < 0.001), whereas total body fat% reduced significantly with −1.5% (fdr < 0.001) in males and −1.5% (fdr < 0.001) in females. In males, lumbar spine BMD increased with 3.0% (fdr < 0.001) and total body BMD with 0.7% (fdr = 0.002). In females, the lumbar spine BMD had a trend in the upwards direction (1.2%, fdr = 0.09) and the total body BMD remained stable (0.4%, fdr = 0.07). In males, the increase in lumbar spine BMD was significantly associated with decreased weight (fdr = 0.001) and with decreased body and trunk fat% (fdr = 0.001, fdr = 0.001) and improved immune‐metabolic health (fdr = 0.02). Males with higher BMD but a poor metabolite‐based health score at baseline had a stronger increase in lumbar spine BMD (fdr = 0.03).

**Conclusions:**

A combined nutritional and activity lifestyle intervention significantly improved BMD of males with good bone health at baseline while at the same time improving metabolic health. Nutritional weight‐loss interventions may not harm BMD when combined with exercise.

## 1 Introduction

Osteoporosis is defined as a chronic disease characterized by low bone mass and deterioration of bone microarchitecture [1]. Bone mass decreases with increasing age, leading to weaker bones susceptible to fractures. Osteoporotic fractures have a major impact on the quality of life, independence and healthcare costs [2]. To maintain healthy bones during ageing, an adequate vitamin D and calcium status is important [3].

One of the risk factors for osteoporotic fractures is a low body mass index (BMI) [4]. Moreover, rapid weight loss leads to a decrease in bone mineral density (BMD) [5, 6]. Weight loss interventions in overweight or obese older adults have shown that weight loss of 8%–10% is accompanied by a substantial decline in BMD of approximately 1.2% in 25 weeks [7], and approximately 2% in 12–24 months [6, 7, 8, 9, 10, 11]. It has been suggested that especially sudden or excessive weight loss in older adults can lead to decreased BMD [12]. One of the possible underlying mechanisms is that weight loss leads to mechanical unloading, which refers to a reduction in the mechanical forces placed on bones [6]. Additionally, if weight loss is accompanied by loss of muscle mass, this increases the risk of falling [13]. If weight loss is caused by a reduced dietary intake, this can lead to an inadequate intake of nutrients that are important for bone health, such as calcium and vitamin D.

When nutritional weight loss is combined with resistance and impact exercise training, adverse effects on BMD seem to be diminished [12, 14]. Exercise causes strains on bones, which stimulates osteocytes (type of bone cells) to produce anabolic factors and stimulate osteoblast activity (responsible for bone formation) [15]. A study in 107 obese older adults (aged > 65 years) demonstrated that a year of caloric restriction with or without a multicomponent exercise training programme led to a comparable weight loss in both groups (diet only: −9.6%, and diet + exercise: −9.4%), whereas the loss in BMD differed (diet only: −2.6%, and diet + exercise: −1.1%) [11]. Similar trends were observed for femoral neck BMD [11]. So, by combining caloric restriction with exercise training, the negative impact of intentional weight loss on BMD can be reduced.

Most weight loss studies are focused on obese adults, have a rather long intervention duration (6 months–1.5 years) [16, 17] and include resistance training. The effect of short‐term lifestyle interventions (shorter than 6 months) on BMD in non‐obese, non‐diabetic, middle‐aged or older adults and improving daily physical activity level, without resistance exercise, next to caloric restriction, is not studied as thoroughly [17]. Besides, most of the studies included a resistance training component, which is known to attenuate the weight loss associated BMD reduction; it is not clear what type of physical activity would be beneficial, feasible and necessary in middle‐aged to older adults. Hence, the interaction between the rate of weight loss and type, frequency and intensity of exercise on BMD changes in non‐obese middle‐aged to older adults needs further investigation.

Previous research has shown that there is a link between metabolic syndrome and low BMD levels, especially relating a higher waist circumference and a higher trunk fat% with lower BMD levels [18, 19]. However, how these effects relate in a healthier population and in the context of a lifestyle intervention has not been studied as thoroughly and requires further exploration.

Here, we investigated changes in total body and lumbar spine BMD in the Growing Old TOgether (GOTO) trial, a 13‐week combined nutritional and physical activity intervention not including resistance exercise in middle‐aged to older adults (aged 46–75 years, BMI level ≥ 23 and ≤ 35 kg/m^2^). In previous research, it has been shown that the GOTO trial improved immune‐metabolic health, reflected by significant changes in weight, BMI, alpha‐1‐acid glycoprotein (GlycA) level, HDL cholesterol level and size, and DEXA‐based body composition measurements [20, 21], part of these effects were related to the sex of the participant. Since the GOTO study was performed without a control group, we additionally investigate the relation of BMD changes with changes in markers of health, including (a) classical diagnostic markers of immuno‐metabolic health (i.e., weight, BMI, serum markers of inflammation, lipid and glucose metabolism), (b) DEXA‐based body composition measurements and (c) novel metabolomics indicators of health previously developed to record immune‐metabolic health (MetaboHealth score) [22] and the metabolomics response to the GOTO trial (by the Personalized Lifestyle Intervention Status [PLIS] score) [23].

The main objectives of the current study were to investigate the effect of a combined nutritional and activity lifestyle intervention on lumbar spine and total body BMD in healthy adults, and to study how these changes relate to body composition and immune‐metabolic health changes.

## 2 Methods

### 2.1 Trial Design

The participants of the GOTO study were non‐diabetic middle‐aged and older adults (mean age 62.9 years). A full description of the recruitment, sample size calculation and inclusion/exclusion criteria of the GOTO study has been published elsewhere [20, 24]. In short, participants were recruited from the Leiden Longevity Study (LLS), a longitudinal cohort containing 421 families with familial longevity defined by at least two long‐living siblings (men aged 89 years or above; women aged 91 years or above) as described in Schoenmaker et al. [25]. The participants performed the lifestyle intervention in couples, generally living in the same household. One of the participants in each couple was recruited from the LLS; the other participant in the couple did not have a known longevity background. At inclusion, the 164 participants had an age between 46 and 75 years, BMI ≥ 23 and ≤ 35 kg/m^2^, and were non‐diabetic. One participant withdrew because of a knee surgery. The reported analyses were performed on a subset of 134 participants (69 males, 65 females) for whom dual‐energy X‐ray absorptiometry (DEXA) data were available (Figure S1).

The Medical Ethical Committee of the Leiden University Medical Center approved the protocol of the GOTO trial prior to the start of the trial (P11.187) and all participants signed a written informed consent. This trial was registered at the Dutch Trial Register (https://onderzoekmetmensen.nl/en) as GOT NL3301 and can also be found at the international clinical trials registry platform as NL‐OMON27183.

The GOTO trial was a 13‐week lifestyle intervention study with 164 healthy middle‐age and older adult participants (49% female, mean ± SD age 62.9 ± 5.7 years, mean ± SD BMI 26.9 ± 2.5 kg/m^2^). The participants underwent a 25% reduction in energy availability, equally divided over 12.5% decreased caloric intake and 12.5% increased physical activity, as described in van de Rest et al. [20]. Before the trial, resting metabolic rate, energy intake and energy expenditure were estimated as described in van de Rest et al. [20]. In short, resting metabolic rate was measured 65 min after a standardized meal by indirect calorimetry, using a ventilated hood system was measured after a standardized meal. Participants were lying on a bed under the ventilated hood in a quiet, temperature‐controlled room for 30 min. The initial 5 min of the measurement were not used for the analysis. VO_2_ and VCO_2_ were measured every minute. Resting energy expenditure (REE) and respiratory quotient (RQ) were calculated using the formulas: *REE = 3.91 VO*_*2*_ *+ 1.10 VCO*_*2*_ *− 1.93N* and *RQ = VCO*_*2*_ */ VO*_*2*_. To determine the baseline energy intake, the habitual dietary pattern of the participants they had to fill in an online food frequency questionnaire (FFQ) [26]. In the FFQ, the participants were asked how often over the past month they have eaten a pre‐specified number of 150 food items. The FFQ was filled at home and subsequently checked for completion and quality of the information by a dietician. The energy expenditure while active was estimated by using the Physical Activity Questionnaire‐Short Form (IPAQ‐SF) [27] in combination with habitual amount of physical activity as measured through an accelerometer (GENEA) on the wrist and ankle. The accelerometer was worn prior to the intervention for a duration of 7 days. Personalized intervention guidelines were prescribed by a dietician and a physiotherapist, in consultation with the participant's preferences and physical abilities. The dietary guidance was composed to be as much as possible according to the ‘Dutch Guidelines for a healthy diet’ [20]. To stimulate better adherence to the lifestyle intervention, participants were advised to increase the amount of physical activity in a way that would match their daily life pattern, with a secondary aim to become fully integrated in the participant's regular daily routine at the end as well as after the intervention. These activities generally included walking, cycling, activities in and around the house and participation in local sport activities, either alone or as a couple.

To stimulate adherence and proper following of the intervention guidelines, the participants had weekly contact with the dietician and physiotherapist, either through telephone, email or at the participant's residence. For each day of the intervention, a participant's dietary and activity compliance to the study programme was self‐reported in an intervention diary (Table S1). The compliance was subsequently summed for each week (ranging from 0 to 7, according to the number of days compliant to the intervention regimen during a given week).

### 2.2 Body Composition and BMD

As described in Beekman et al. [21], data of seven anthropometrics based on weight, height, waist and hip circumference were available. Body composition was measured using DEXA (Discovery A, Hologic Inc., Bedford, MA, USA), including total body lean mass, total body fat in percentage of total body weight and percentage of trunk fat to total trunk weight. In addition, BMD measurements were available for total body BMD and lumbar spine BMD in g/cm^2^. The Z‐score of the total body BMD was calculated using the reference values described in Kelly et al. [28].

### 2.3 Biomarkers

Biomarkers representing lipid metabolism, inflammation and those relevant for BMD from the IGF and vitamin D pathways were selected. All measurements were performed in fasting serum collected through venipuncture. HDL cholesterol, HDL cholesterol size and the novel inflammation markers GlycA were measured using the previously described Nightingale hydrogen‐1 nuclear magnetic resonance (^1^H‐NMR) metabolite platform [20, 29]. Fasting insulin‐like growth factor 1 (IGF‐1) and insulin‐like growth factor binding protein 3 (IGFBP‐3) levels were measured using an Immulite 2000 XPi (Siemens, Eschborn, Germany). Fasting serum 25‐hydroxy vitamin D (25(OH)D) levels were measured using the Roche Cobas Vitamin D total kit.

### 2.4 Novel Indicators of Health: Metabolomics Scores

To assess the immune‐metabolic health status of the participants, we calculated a previously developed metabolomic score (MetaboHealth) [22] and a metabolomics‐based intervention monitoring score (PLIS) [23].

The MetaboHealth score was trained on 44 168 individuals from 12 different cohorts and is significantly positively associated with all‐cause mortality. This score uses 14 fasting serum ^1^H‐NMR metabolite levels: total lipids in chylomicrons and extremely large VLDL, total lipids in small HDL, mean diameter for VLDL particles, ratio of polyunsaturated fatty acids to total fatty acids (%), glucose, lactate, histidine, isoleucine, leucine, valine, phenylalanine, acetoacetate, albumin and GlycA [22] and is considered a marker of immune‐metabolic health. A high MetaboHealth score represents poor immune‐metabolic health status as reflected by its association to increased risk of cognitive decline, frailty and mortality [22, 30, 31].

The PLIS score was trained on the participants of the GOTO study and aimed to capture the heterogeneity of the response to the intervention. The PLIS score was trained in a sex‐stratified manner and was optimized to capture the metabolic health response to a combined lifestyle intervention. The male PLIS score is composed of four fasting serum ^1^H‐NMR metabolite levels: histidine, citrate, total lipids in small VLDL and the ratio of saturated fatty acids to total fatty acids (%). The female PLIS score is composed of 14 fasting serum ^1^H‐NMR metabolite levels: glutamine, histidine, phenylalanine, tyrosine, leucine, glucose, citrate, 3‐hydroxybutyrate, creatinine, total lipids in chylomicrons and extremely large VLDL, sphingomyelins, apolipoprotein A1, docosahexaenoic acid and the ratio of saturated fatty acids to total fatty acids (%) [23]. An increase in the PLIS score represents a change to a healthier metabolic health profile.

### 2.5 Statistical Analyses

To investigate the difference between baseline characteristics between male and female participants, a two‐sided unpaired *t*‐test was used.

The association analysis between baseline BMD and health markers was performed using a two‐sided linear model analysis, by using the *lm* function of the R package *stats* (Version 4.2.2). Prior to the analysis, both the BMD and the biomarker levels and immune‐metabolic health score levels were z‐scaled (mean of 0 and standard deviation of 1). Age at baseline was added as a fixed effect.

The effect of the intervention on bone health and other health markers was calculated using a two‐sided linear mixed model analysis, the *lmer* function from the R package *lmerTest* (Version 3.1.3); age at baseline was added as a fixed effect and person id as a random effect. To calculate the Hedges' g effect size, the function *cohen.d* was used from the R package *effsize* (Version 0.8.1).

The effect of total lean mass on the intervention effect on total body BMD and lumbar spine BMD was studied using a causal inference analysis. The causal inference analysis was performed using the *mediate* function from the R package *mediation* (Version 4.5.1).

With the exception of the analyses performed at baseline, all analyses were performed in a paired approach, thus comparing a participant's post intervention measurement to their respective baseline measurement. All analyses were performed in a sex‐stratified manner. When applicable, the significance levels were adjusted for multiple testing using the Benjamini–Hochberg false discovery rate (FDR) correction; an FDR‐adjusted *p*‐value below 0.05 was considered significant.

## 3 Results

### 3.1 Sex Differences in Baseline Characteristics of GOTO Participants

At baseline, BMD, biomarkers, body composition and metabolic health scores were measured (Table 1). Of these 16 measurements, 11 were significantly different at baseline between males and females, including total body (fdr < 0.001) and lumbar spine BMD (fdr = 0.001), which were both higher in males than in females. The total body BMD derived Z‐score (see Section 2) was also trending higher in male participants, but not significantly (fdr = 0.23). For these reasons, we have analysed the intervention effect for male and female participants separately.

**TABLE 1:** Baseline characteristics of GOTO participants with DEXA scan measurements by sex.

Health marker	Males		Females		*p*‐adjust
	*n*	Mean (SD) [limits]	*n*	Mean (SD) [limits]	
Age, years	69	63.8 (5.5) [50.0–73.7]	65	61.9 (6.3) [46.7–75.1]	0.13
Bone mineral density					
Lumbar spine BMD, g/cm^2^	69	1.1 (0.2) [0.7–1.5]	64	1.0 (0.2) [0.6–1.4]	0.004
Total body BMD, g/cm^2^	69	1.2 (0.1) [0.9–1.4]	65	1.0 (0.1) [0.8–1.3]	< 0.001
Z‐score	69	0.1 (1.1) [−2.2 to 2.0]	65	−0.2 (1.0) [−3.4 to 1.7]	0.23
Biomarkers					
Fasting HDL cholesterol, mmol/L	67	1.4 (0.2) [0.8–2.0]	64	1.7 (0.3) [1.1–2.3]	< 0.001
Fasting HDL cholesterol size, nm	66	9.5 (0.1) [9.2–9.7]	65	9.6 (0.2) [9.3–10.1]	< 0.001
Fasting GlycA, mmol/L	66	0.8 (0.1) [0.6–1.0]	64	0.8 (0.1) [0.7–1.1]	0.62
Fasting IGF‐1, nmol/L	69	21.4 (4.8) [7.8–31.9]	64	18.6 (4.8) [8.4–29.9]	0.002
Fasting IGFBP‐3, mg/L	67	3.8 (0.7) [1.7–5.3]	65	4.2 (0.9) [2.2–6.7]	0.007
Fasting 25(OH)D, nmol/L	69	69.8 (27.1) [14.8–147.7]	64	63.4 (25.6) [14.1–125.1]	0.23
Body composition					
Weight, kg	69	84.1 (8.2) [67.1–104.9]	65	74.1 (8.9) [60.4–100.4]	< 0.001
BMI, kg/m^2^	68	26.6 (2.2) [22.5–34.0]	65	27.2 (2.9) [23.0–33.7]	< 0.001
Total body fat, %	69	26.4 (4.1) [15.9–34.9]	65	38.3 (4.4) [27.8–46.2]	< 0.001
Trunk fat, %	69	28.1 (5.2) [12.7–38.5]	65	37.4 (5.7) [22.2–49.0]	< 0.001
Total lean mass, kg	69	62.1 (6.0) [49.0–76.7]	63	45.7 (4.4) [37.5–59.1]	< 0.001
Metabolite‐based health scores					
MetaboHealth	64	−0.1 (0.3) [−1.0 to 0.8]	61	0.0 (0.3) [−0.8 to 0.8]	0.64
Personalized Lifestyle Intervention Status	62	0.5 (0.1) [0.3–0.7]	62	0.5 (0.2) [0.0–1.0]	0.76

*Note:* *p*‐adjust represents the Benjamini–Hochberg fdr‐adjusted significance level of the difference between males and females at baseline. Significance levels were determined using a two‐sided unpaired *t*‐test.

Abbreviations: 25(OH)D, 25‐hydroxy vitamin D; BMD, bone mineral density; BMI, body mass index; GlycA, alpha‐1‐acid glycoprotein; HDL, high‐density lipoprotein; IGF‐1, insulin‐like growth factor 1; IGFBP‐3, insulin‐like growth factor binding protein 3; SD, standard deviation.

### 3.2 Intervention Effect on Lumbar Spine and Total Body BMD

**FIGURE 1:**
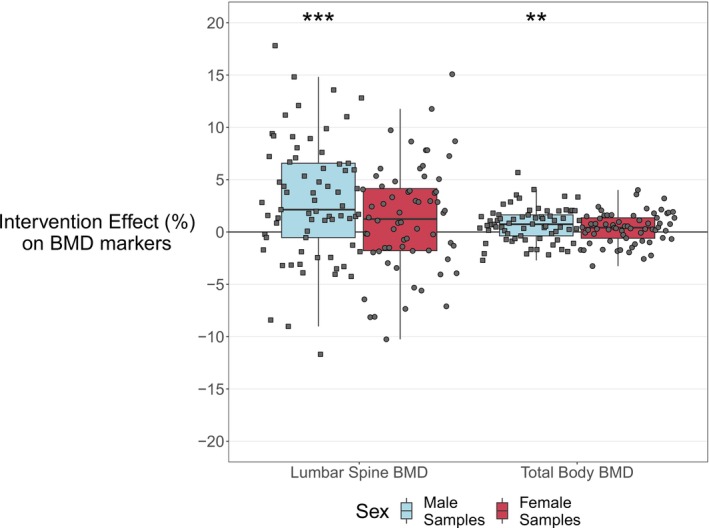
The 13‐week lifestyle intervention effects on lumbar spine and total body BMD in males and females. The x‐axis shows the different BMD measurements, and the y‐axis the personalized percentage of change (%) with respect to their measurement at the start of intervention (baseline). The light‐blue boxplot represents the results of males, the dark red boxplot those of females. Significance is indicated by the asterisks (* = BH fdr < 0.05; ** = BH fdr < 0.01, *** = BH fdr < 0.001). BH, Benjamini–Hochberg; BMD, bone mineral density.

The intervention significantly increased BMD of the lumbar spine and total body, and the total body BMD Z‐score in male participants, while in female participants the lumbar spine had an upward trend and the total body BMD remained stable (Table 2, Figure 1). The mean percentage of lumbar spine change was +3.0% (SD 5.7, fdr < 0.001) and +1.2% (SD 5.0, fdr = 0.09) for males and females, respectively. For total body BMD, the changes were +0.7% (SD 1.6, fdr = 0.002) for males and +0.4% (SD 1.5, fdr = 0.07) for females. The total body BMD Z‐score increased by 0.07 (SD 0.2, fdr < 0.001) in male participants, while in the female participants, there was a weak upward trend of 0.04 (SD 0.2, fdr = 0.07).

**TABLE 2:** Intervention effect on health markers.

	Males					Females				
	*n*	Mean delta (SD)	Mean % of change (SD)	Hedges' g	*p*‐adjust	*n*	Mean delta (SD)	Mean % of change (SD)	Hedges' g	*p*‐adjust
Bone mineral density										
Lumbar spine BMD, g/cm^2^	69	0.03 (0.06)	+3.0 (5.7)	0.16	< 0.001	64	0.01 (0.05)	+1.2 (5.0)	0.05	0.09
Total body BMD, g/cm^2^	69	0.008 (0.02)	+0.73 (1.6)	0.07	0.002	65	0.004 (0.02)	+0.36 (1.5)	0.04	0.07
Z‐score	69	0.07 (0.2)	—	0.07	< 0.001	65	0.04 (0.2)	—	0.04	0.07
Biomarkers										
Fasting HDL cholesterol, mmol/L	67	0.04 (0.1)	+3.4 (10.1)	0.15	0.08	64	−0.03 (0.1)	−1.1 (7.9)	−0.10	0.13
Fasting HDL cholesterol size, nm	66	0.06 (0.09)	+0.6 (0.96)	0.39	< 0.001	65	0.03 (0.08)	+0.29 (0.8)	0.16	0.01
Fasting GlycA, mmol/L	66	−0.02 (0.07)	−2.5 (8.7)	−0.28	0.02	64	−0.02 (0.06)	−2.0 (6.9)	−0.19	0.03
Fasting IGF‐1, nmol/L	68	0.3 (2.9)	+2.5 (14)	0.08	0.41	64	−0.2 (3.1)	+0.43 (17.4)	−0.02	0.68
Fasting IGFBP‐3, mg/L	66	0.01 (0.5)	+0.95 (14.1)	−0.09	0.73	65	−0.07 (0.6)	−0.14 (14.6)	−0.08	0.41
Fasting 25(OH)D, nmol/L	68	−7.6 (13.9)	−10.2 (20.9)	−0.26	< 0.001	64	−3.3 (14.0)	−5.5 (24.7)	−0.11	0.09
Body composition										
Weight, kg	69	−3.3 (2.5)	−3.9 (3)	−0.40	< 0.001	65	−3.4 (2.2)	−4.6 (2.9)	−0.37	< 0.001
BMI, kg/m^2^	69	−1.0 (0.8)	−3.9 (3)	−0.46	< 0.001	65	−1.2 (0.8)	−4.6 (2.9)	−0.43	< 0.001
Total body fat, %	69	−1.5 (1.7)	−5.9 (6.4)	−0.33	< 0.001	65	−1.5 (1.7)	−4.1 (4.9)	−0.29	< 0.001
Trunk fat, %	69	−2.3 (2.1)	−8.7 (7.8)	−0.41	< 0.001	65	−2.1 (2.2)	−6.0 (6.4)	−0.32	< 0.001
Total lean mass, kg	69	−1.4 (1.2)	−2.2 (1.9)	−0.23	< 0.001	64	−1.1 (1.2)	−2.3 (2.5)	−0.23	< 0.001
Metabolite‐based health scores										
MetaboHealth	63	0.07 (0.4)	—	0.18	0.09	60	0.09 (0.3)	—	0.25	0.04
Personalized Lifestyle Intervention Status	62	0.06 (0.09)	+15 (21.1)	0.65	< 0.001	62	0.07 (0.2)	+28.2 (65.2)	0.38	0.002

*Note:* Mean delta represents the mean effect of the intervention. Mean % of change represents the participant's change relative to their baseline value. *p*‐adjust represents the Benjamini–Hochberg fdr‐adjusted significance level of the intervention effect. Significance level was determined through a two‐sided linear mixed model, adjusted for age at baseline as a fixed effect and person ID as a random effect. Since the Z‐score and the MetaboHealth have both positive and negative values, the % change of these variables has not been calculated.

Abbreviations: 25(OH)D, 25‐hydroxy vitamin D; BMD, bone mineral density; BMI, body mass index; HDL, high‐density lipoprotein; IGF‐1, insulin‐like growth factor 1; IGFBP‐3, insulin‐like growth factor binding protein 3; SD, standard deviation.

### 3.3 Intervention Effect on Overall Health

The weight of the 134 participants was significantly reduced (−3.3 kg, SD 2.5, fdr < 0.001 [−3.9%, SD 3%] and −3.4 kg, SD 2.2 [−4.6%, SD 2.9%]) for males and females, respectively, as well as BMI (−1.0 kg/m^2^, SD 0.8, fdr < 0.001 and −1.2 kg/m^2^, SD 0.8, fdr < 0.001), total body fat% (−1.5, SD 1.7, fdr < 0.001 and −1.5, SD 1.7, fdr < 0.001) and trunk fat% (−2.3, SD 2.1, fdr < 0.001 and −2.1, SD 2.2, fdr < 0.001), and total lean mass (−1.4 kg, SD 1.2, fdr < 0.001 and −1.1 kg, SD 1.2, fdr < 0.001) (Table 2). Notably, even though the total lean mass reduced in both sexes, the lean mass percentage increased in both sexes with 1.5% (male SD 1.7, female SD 1.7), indicating that the participants had a bigger reduction of fat mass than lean mass. The male and female participants also improved their immune‐metabolic health as reflected by the significant increase in fasting HDL cholesterol size (0.06 nm, SD 0.09, fdr < 0.001 and 0.03 nm, SD 0.08, fdr = 0.01) and fasting GlycA levels (−0.02 mmol/L, SD 0.07, fdr = 0.02 and −0.02 mmol/L, SD 0.06, fdr = 0.03) (Table 2). Fasting 25(OH)D level reduced significantly in males (−7.6 nmol/L, SD 13.9, fdr < 0.001) and was trending in the downwards direction in females (−3.3 nmol/L, SD 14.0, fdr = 0.09), which was linked to the season in which participants started (Figure S3). The intervention did not have an effect on the factors reflecting the IGF pathway. Lastly, of the two composite metabolite‐based health scores, the MetaboHealth increased significantly in female participants (0.09, SD 0.3, fdr = 0.04), but not in males (0.07, SD 0.4, fdr = 0.09), whereas the PLIS score increased significantly both in males (0.06, SD 0.09, fdr < 0.001) and females (0.07, SD 0.2, fdr = 0.002). The effect of the intervention on different health markers was to the same extent as the original complete set of 163 participants, as reported by Van de Rest et al. [20].

### 3.4 Intervention Effect on BMD Measurements Was Independent From Total Lean Mass

To investigate whether the effect of the intervention on total body BMD and lumbar spine BMD was influenced by total lean mass, a mediation analysis was performed (see Section 2). Since only the male participants significantly increased their BMD levels, the mediation analysis was only performed in male participants. The effects of the intervention on the lumbar spine BMD (average causal mediated effect [ACME] = −0.002, fdr = 0.7) and the total body BMD (ACME = −0.004, fdr = 0.18) were independent from the effect of the intervention on total lean mass.

### 3.5 Baseline Associations Between BMD and Health Markers

**FIGURE 2:**
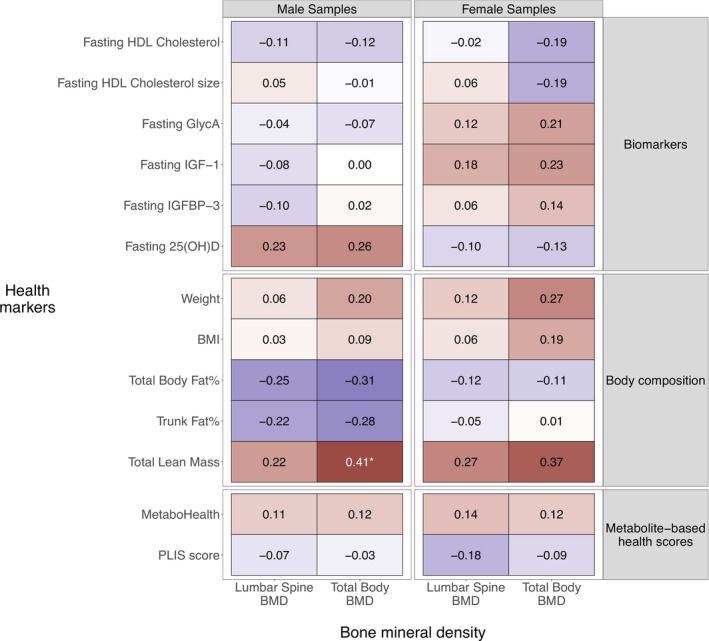
Association between BMD and health markers at baseline. Cells represent the association (based on a linear effect model, see Section 2) between the different BMD measurements (columns) and the different health markers (rows). The colour represents the direction of the estimated effects: red, a positive association; blue, a negative association; white, no association. Significance of the associations is indicated by the asterisks (* = BH fdr < 0.05). 25(OH)D, 25‐hydroxy vitamin D; BH, Benjamini–Hochberg; BMD, bone mineral density; BMI, body mass index; GlycA, alpha‐1‐acid glycoprotein; HDL, high‐density lipoprotein; IGF‐1, insulin‐like growth factor 1; IGFBP‐3, insulin‐like growth factor‐binding protein 3.

In males, the only significant association at baseline was between total body BMD and total lean mass with an estimated effect size of 0.41 (fdr = 0.03) (linear model, see Section 2), meaning that participants with a 1 SD higher total lean mass at baseline had a 0.41 SD higher total body BMD (Figure 2). The same trend was observed for weight (0.20, fdr = 0.34) and BMI (0.09, fdr = 0.76). DEXA‐based total body (−0.31, fdr = 0.18) and trunk fat% (−0.28, fdr = 0.24) showed a negative trend with lumbar spine and total body BMD although again not significant. Finally, 25(OH)D seemed to trend positively with both lumbar spine BMD (0.23, fdr = 0.30) and total body BMD (0.26, fdr = 0.26). The remaining biomarkers, including the two metabolic health scores, MetaboHealth (0.11, fdr = 0.66 and 0.12, fdr = 0.65) and the PLIS score (−0.07, fdr = 0.81 and −0.03, fdr = 0.92), were not associated with both lumbar spine and total body BMD, respectively.

In females, there were no significant associations between BMD and health markers after multiple testing correction. The strongest trend was between total body BMD with total lean mass (0.37, fdr = 0.09) and weight (0.27, fdr = 0.24). The biomarkers fasting GlycA (0.21, fdr = 0.30), IGF‐1 (0.23, fdr = 0.30) and IGFBP‐3 (0.14, fdr = 0.59) were also trending positively to total body BMD, whereas HDL cholesterol level (−0.19, fdr = 0.35) and size (−0.19, fdr = 0.35) showed a negative trend with total body BMD.

### 3.6 Associations Between Intervention Effect on BMD and Health Markers Over Time

To investigate how the effects of the intervention on different measurements related to each other, we investigated the association between BMD and the different health markers across both time points of the intervention.

**FIGURE 3:**
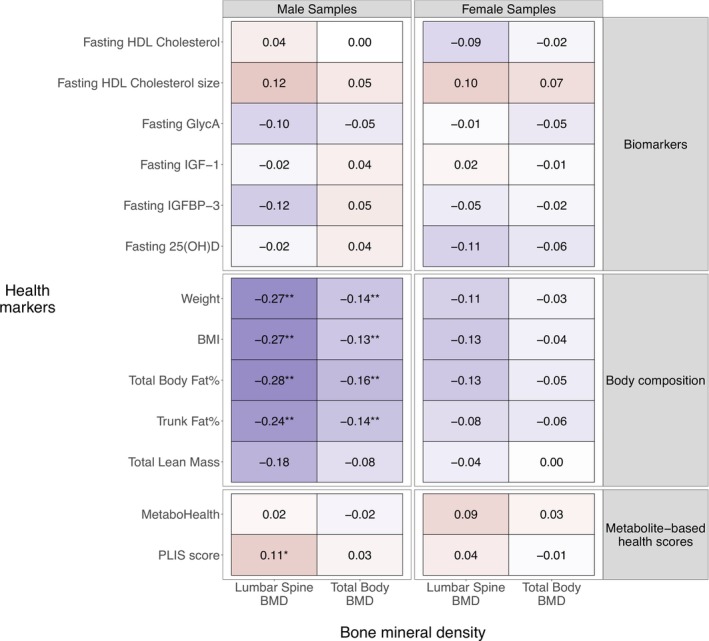
Association between BMD and health markers across both time points of the intervention. Cells represent the association (based on a linear mixed effect model, see Section 2) between the different BMD measurements (columns) and the different health markers (rows). The colour represents the direction of the estimated effects: red, a positive association; blue, a negative association; white, no association. Significance of the associations is indicated by the asterisks (* = BH fdr < 0.05, ** = BH fdr < 0.01). 25(OH)D, 25‐hydroxy vitamin D; BH, Benjamini–Hochberg; BMD, bone mineral density; BMI, body mass index; GlycA, alpha‐1‐acid glycoprotein; HDL, high‐density lipoprotein; IGF‐1, insulin‐like growth factor 1; IGFBP‐3, insulin‐like growth factor‐binding protein 3.

In males, changes in lumbar spine and total body BMD were negatively associated with changes in weight (−0.27, fdr = 0.001 and −0.14, fdr = 0.004), BMI (−0.27, fdr = 0.001 and −0.13, fdr = 0.001) and DEXA‐based total body fat% (−0.28, fdr = 0.001 and −0.16, fdr = 0.001) and trunk fat% (−0.24, fdr = 0.002 and −0.14, fdr = 0.001) (Figure 3). Additionally, there was a significant positive association between lumbar spine BMD and the PLIS score (0.11, fdr = 0.02). The remaining associations were not significant.

In females, none of the associations were significant after multiple testing correction. However, in terms of direction of effect between the BMD and health markers, the results were similar to those in males but weaker.

### 3.7 Baseline Metabolic Health and Change in BMD

**FIGURE 4:**
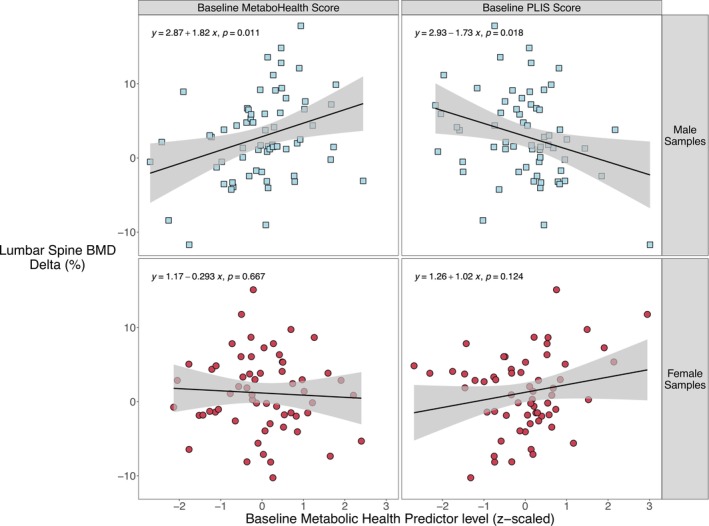
The association of lumbar spine BMD percentage change with baseline metabolic health scores. The x‐axis represents the z‐scaled baseline metabolic health scores. The y‐axis represents the percentage of change in lumbar spine BMD. The line through the data represents the estimated effect between the reported percentage of change in lumbar spine BMD and the baseline metabolic health score, the grey ribbon represents the 95% confidence interval. The formula of the estimated effect and the nominal significance level are plotted in the top‐left corner of each panel.

Lastly, we investigated whether the baseline immune‐metabolic health status assessed with the MetaboHealth score and the PLIS score could be indicators of the strength of the BMD response to the intervention. In males, a lower immune‐metabolic health status at baseline (i.e., represented by a high MetaboHealth score and a low PLIS score) was significantly associated with a stronger lumbar spine BMD response (% delta), with an estimated effect of 1.82 (fdr = 0.03) and −1.73 (fdr = 0.03), for the MetaboHealth and PLIS score, respectively (Figure 4). Meaning that for each SD higher MetaboHealth at baseline, there was a 1.82% increase in lumbar spine BMD. And for each SD lower baseline PLIS score, the improvement of lumbar spine BMD was 1.73% higher.

In females, there was no significant association between baseline metabolic health status and percentage of change in BMD.

These results indicate that male participants with a lower immune‐metabolic health status (a high MetaboHealth score or a low PLIS score) had a larger improvement of their lumbar spine BMD with respect to their baseline levels than males with more healthy scores. This was observed despite the fact that no association existed between the baseline immune‐metabolic health status and the baseline lumbar spine BMD levels (Figure S2).

## 4 Discussion

This study found that a 13‐week combined nutritional and activity lifestyle intervention (GOTO trial, trial registration number GOTNL3301 (https://onderzoekmetmensen.nl/nl/trial/27183), NL‐OMON27183) significantly increased the BMD in the lumbar spine (+3.0%) and the total body (+0.7%) in males, with a significant total body BMD Z‐score increase of 0.07. In females, the BMD levels remained stable. At baseline, total body BMD was significantly positively associated with total lean mass in male participants. During the intervention, in males, an increase in BMD was significantly associated with reduced weight, total body fat%, trunk fat%, increased HDL cholesterol and improved immune‐metabolic health, as indicated by the PLIS score. Lastly, males with poor immune‐metabolic health at baseline showed a stronger increase in their lumbar spine BMD during the intervention.

Previously, it was found that weight loss interventions are accompanied by a substantial decline in BMD [6, 12]. However, the results of our study indicate that intentional weight loss in middle‐aged to older adults seems harmless for BMD, possibly because the weight loss is induced not only by the diet but also by exercise training. While there are multiple lifestyle intervention studies in obese middle‐aged and older adults [16, 17, 32], studies investigating the effect of such an intervention on BMD in healthy middle‐aged to older adults are scarce, making comparison of results difficult. A 1‐year study in 107 obese older adults (aged > 65 years) found a faster decline in total hip BMD by dietary caloric restriction (−2.6%) than in the group which combined diet and exercise to lose weight (−1.1%) [11]. In a small weight‐loss study with older, overweight adults with type 2 diabetes (*n* = 36, age 60–80 years), BMD remained stable in the diet + exercise group after 6 months, while it was decreased in the diet group only [32]. In another small study in healthy, postmenopausal women (*n* = 30, mean age 63) [33], both the diet group and diet + exercise group experienced total body BMD loss after 6 months. However, BMD loss was more substantial in the diet group. BMD in the hip also decreased in the diet group but not in the combined group. In 2016, a systematic review and meta‐analysis was conducted focused on the effects of 32 weight loss strategies, including caloric restriction and exercise training, on BMD in adults (mean age ranging from 25 to 77 years) [34]. Studies with caloric restriction only showed a decline in BMD (mean delta [MD] total body −0.003 g/cm^2^, spine MD −0.020 g/cm^2^), whereas studies with only an exercise programme did not (total body MD 0.001 g/cm^2^, spine MD 0.008 g/cm^2^). The same systematic review stated that a combination of caloric restriction with an exercise programme had a significant positive effect on total body BMD (MD 0.004 g/cm^2^, spine MD), but no effect on spine BMD (MD 0.00 g/cm^2^) [34]. These results are largely in line with the current study, where even larger effect sizes were found (in males MD 0.03 g/cm^2^ for lumbar spine BMD and MD 0.01 g/cm^2^ for total body BMD).

It is remarkable that the effects on BMD already occurred after 13 weeks in the current study. In general, a longer duration of exposure is needed to induce changes in BMD. However, in the study of Hill et al., a significant increase in total body BMD was seen after consuming a vitamin D, calcium and protein supplement for 13 weeks in sarcopenic non‐malnourished older adults (*n* = 129, mean age 78) [35].

In the current trial, the larger effect on lumbar spine BMD compared to total body BMD can be explained. Namely, spine BMD contains cancellous bone, which is often more responsive to stimuli than cortical bone [36]. The difference in effect between males and females is more difficult to explain. BMD at the start of the study was lower in females than in males. In addition, it is known that the age‐related decline in BMD is stronger in females than in males [37], which could mean that females require a more intense and/or longer exercise intervention. However, in a smaller subset of the GOTO study, we did notice some differences between the activity patterns among male and female participants; including that male participants showed a trend in having higher activity levels measured at the ankle and in walking more than the female participants [38]. Walking is a weight‐bearing activity that can stimulate bone formation [39]. Lastly, multiple variables were different at baseline between the sexes; total lean mass and weight were both significantly higher in males, which can also influence BMD.

The 134 participants improved their metabolic health and significantly reduced weight, BMI and total body fat%. Contrary to the BMD effects, in both male and female participants, the fasting 25(OH)D levels reduced during the intervention, with significant effects in males. This decrease in fasting 25(OH)D can be explained by the starting dates of the intervention, which was in autumn or early winter for the majority of the participants (108/134) (Figure S3). Hence, we postulate that the effects were mainly seasonal. When investigating the association between baseline BMD and baseline health markers, only total body BMD and total lean mass were significantly positively associated in males. No other significant associations were found after correcting for multiple testing in either males or females. However, we did identify a striking difference between the estimated effect sizes of the two BMD measurements and fat% measurements, which were strongly negative in males and only weakly negative in females (Figure 2). This difference might be explained by the difference of fat% levels at baseline between the two sexes (Table 1), due to a difference in fat between males and females or the effect sex hormones play on both the fat mass distribution and BMD turnover [36].

When studying the associations across both time points of the intervention trial, several weight‐related health markers were significantly inversely associated with both lumbar spine and total body BMD; these effects were only significant in males. Additionally, there was a significant positive association between the PLIS score and lumbar spine BMD (0.11). These results indicate that the participants who had a greater improvement of immune‐metabolic health also showed larger lumbar spine BMD level improvements. At first, losing weight and body fat while gaining BMD might appear counterintuitive, since weight loss interventions generally reduce BMD level [8, 12]. However, when weight loss interventions contain resistance training or high impact components, this loss can be attenuated [8, 11, 12, 16, 17]. From the results of the GOTO study, we can now postulate that even a combined intervention with moderately reducing caloric intake with concurrently increasing habitual physical activity levels is sufficient to either improve or stop the decline of the BMD levels. Lastly, baseline immune‐metabolic health status (as assessed with the MetaboHealth score and the PLIS score) was an indicator of the strength of the BMD response to the intervention in males. A significant association was found between a lower metabolic health status at baseline and a higher percentage‐based lumbar spine BMD improvement during the intervention. In an earlier study we showed that participants of the GOTO study with a lower PLIS score benefited more from the intervention in their metabolic health improvement [23]. Since these participants had a greater improvement in their metabolic health, most likely due to their poor baseline metabolic health status, and the improvement of lumbar spine was significantly associated to the improved metabolic health status, we postulate that the greater lumbar spine BMD improvement was a by‐product of the metabolic health improvement. However, to better understand the possible mechanism behind this phenomenon, the relationship between metabolic health improvements and BMD improvements needs to be studied further.

A limitation of this trial includes the absence of a control group, which makes it difficult to draw definitive conclusions about the causal relationship between the intervention and the observed results. In addition, the lack of data on the menopausal status is a limitation of the study, especially considering the age range of the female participants (46–75). Moreover, the menopausal status is known to have a large effect on the BMD level [40], which could be related to the overall weaker BMD effect in female participants compared to male participants. To investigate whether this is the case, future studies with similar characteristics to GOTO should control for menopausal status to ensure that sexual dimorphism is appropriately explored before ruling out the possibility of benefits of this intervention in females.

Overall, we showed that a mild catabolic combined lifestyle intervention of 13 weeks increased lumbar spine and total body BMD in 134 healthy middle‐aged to older adults, with significant increases in males. We believe that the results of this trial can be informative on how to design weight‐loss interventions that do not harm the total body and lumbar spine BMD levels. Note that the results of this trial cannot be extrapolated to older adults with osteopenia or osteoporosis. In these persons, BMD is already low or within critical limits. Thereby, caution is warranted to follow a weight‐loss intervention, which can potentially lower their BMD even further.

## Funding

This work was funded by the Netherlands Consortium for Healthy Ageing (NWO grant 050‐060‐810), within the framework of the BBMRI Metabolomics Consortium funded by BBMRI‐NL (NWO 184.021.007 and 184.033.111) and ZonMw Project VOILA (ZonMW 457001001). The funding agencies had no role in the design and conduct of the trial; collection, management, analysis and interpretation of the data; and preparation, review or approval of the manuscript.

## Conflicts of Interest

The authors declare no conflicts of interest.

## Supporting information


**Table S1:** Compliance data of the GOTO participants with DEXA measurements.
**Figure S1:** Flowchart of the participants screening and selection for the Growing Old TOgether study.
**Figure S2:** Association of baseline lumbar spine BMD and baseline health marker scores. On the x‐axis, the z‐scaled baseline metabolic health score is plotted. On the baseline lumbar spine BMD is plotted. The line through the data represents the estimated effect between the lumbar spine BMD change and the baseline metabolic health score, the grey ribbon represents the 95% confidence interval. The formula of the estimated effect and the significance level are plotted in the top‐left corner of each panel.
**Figure S3:** Fasting vitamin D levels at baseline and post intervention, plotted per month of intervention starting date. X‐axis represents the baseline fasting vitamin D levels. Y‐axis represents the post intervention fasting vitamin D levels. Blue squares represent male samples, red circles represent female samples. Months indicate the starting month of the intervention.

## Data Availability

The data that support the findings of this trial are accessible upon request to the corresponding author. The data are not publicly available due to privacy or ethical restrictions. The protocol of the GOTO lifestyle intervention trial is accessible upon request to Marian Beekman (m.beekman@lumc.nl).
