# Supplementary material for: Effects of a Combined Dietary and Physical Activity Intervention on Bone Density, Lean Mass and Fat Mass in Adults: The GOTO Trial
Source: J Cachexia Sarcopenia Muscle. 2026 Mar 8;17(2):e70226. doi: 10.1002/jcsm.70226 (PMC12968446; doi:10.1002/jcsm.70226)
Supplement: Supplementary file 1 — Table S1: Compliance data of the GOTO participants with DEXA measurements. Figure S1: Flowchart of the participants screening and selection for the Growing Old TOgether study. Figure S2: Association of baseline lumbar spine BMD and baseline health marker scores. On the x‐axis, the z‐scaled baseline metabolic health score is plotted. On the baseline lumbar spine BMD is plotted. The line through the data represents the estimated effect between the lumbar spine BMD change and the baseline metabolic health score, the grey ribbon represents the 95% confidence interval. The formula of the estimated effect and the significance level are plotted in the top‐left corner of each panel. Figure S3: Fasting vitamin D levels at baseline and post intervention, plotted per month of intervention starting date. X‐axis represents the baseline fasting vitamin D levels. Y‐axis represents the post intervention fasting vitamin D levels. Blue squares represent male samples, red circles represent female samples. Months indicate the starting month of the intervention. [file JCSM-17-e70226-s001.docx]

# Supplementary Tables

Supplementary Table 1: Compliance data of the GOTO participants with DEXA measurements.

| person id | compliance type | sex | wk1 | wk2 | wk3 | wk4 | wk5 | wk6 | wk7 | wk8 | wk9 | wk10 | wk11 | wk12 | wk13 | mean |
| --- | --- | --- | --- | --- | --- | --- | --- | --- | --- | --- | --- | --- | --- | --- | --- | --- |
| 1 | dietary | male | 6 | 5 | 6 | 6 | 5 | 5 | 5 | 7 | 4 | 5 | 5 | 5 | 2 | 5.08 |
| 1 | activity | male | 7 | 6 | 7 | 6 | 4 | 7 | 6 | 6 | 4 | 7 | 6 | 5 | 2 | 5.62 |
| 2 | dietary | male | 6 | 6 | 5 | 7 | 6 | 7 | 4 | 4 | 6 | 7 | 4 | 6 | 0 | 5.23 |
| 2 | activity | male | 5 | 5 | 7 | 7 | 6 | 5 | 3 | 6 | 4 | 6 | 3 | 6 | 0 | 4.85 |
| 3 | dietary | male | 6 | 6 | 6 | 7 | 7 | 7 | 7 | 7 | 7 | 7 | 7 | 7 | 1 | 6.31 |
| 3 | activity | male | 6 | 6 | 7 | 6 | 6 | 7 | 7 | 7 | 7 | 7 | 7 | 7 | 1 | 6.23 |
| 4 | dietary | male | 5 | 5 | 6 | 5 | 6 | 6 | 3 | 7 | 7 | 4 | 6 | 5 | 1 | 5.08 |
| 4 | activity | male | 7 | 6 | 6 | 5 | 6 | 6 | 7 | 6 | 4 | 4 | 5 | 6 | 1 | 5.31 |
| 5 | dietary | male | 4 | 0 | 5 | 7 | 3 | 0 | 0 | 0 | 0 | 0 | 0 | 0 | 0 | 1.46 |
| 5 | activity | male | 1 | 0 | 5 | 7 | 0 | 0 | 0 | 0 | 0 | 0 | 0 | 0 | 0 | 1 |
| 6 | dietary | male | 4 | 6 | 6 | 5 | 5 | 6 | 5 | 6 | 7 | 5 | 5 | 4 | 0 | 4.92 |
| 6 | activity | male | 5 | 7 | 6 | 5 | 6 | 7 | 5 | 5 | 7 | 6 | 4 | 4 | 0 | 5.15 |
| 7 | dietary | male | 6 | 5 | 6 | 6 | 5 | 5 | 7 | 6 | 4 | 7 | 6 | 6 | 0 | 5.31 |
| 7 | activity | male | 7 | 5 | 6 | 4 | 4 | 4 | 5 | 3 | 5 | 5 | 5 | 5 | 0 | 4.46 |
| 8 | dietary | male | 7 | 7 | 7 | 7 | 7 | 6 | 7 | 7 | 7 | 7 | 7 | 7 | 0 | 6.38 |
| 8 | activity | male | 6 | 6 | 3 | 3 | 5 | 2 | 2 | 4 | 5 | 4 | 2 | 2 | 0 | 3.38 |
| 9 | dietary | male | 5 | 5 | 5 | 4 | 5 | 6 | 5 | 6 | 7 | 5 | 3 | 6 | 5 | 5.15 |
| 9 | activity | male | 7 | 7 | 7 | 7 | 7 | 4 | 1 | 4 | 6 | 5 | 7 | 7 | 6 | 5.77 |
| 10 | dietary | male | 4 | 3 | 2 | 2 | 5 | 5 | 3 | 6 | 4 | 4 | 4 | 4 | 3 | 3.77 |
| 10 | activity | male | 7 | 7 | 7 | 7 | 7 | 7 | 7 | 7 | 6 | 7 | 7 | 7 | 3 | 6.62 |
| 11 | dietary | male | 7 | 6 | 7 | 5 | 6 | 6 | 6 | 7 | 0 | 4 | 5 | 6 | 0 | 5 |
| 11 | activity | male | 3 | 2 | 4 | 3 | 5 | 3 | 3 | 4 | 2 | 4 | 5 | 6 | 0 | 3.38 |
| 12 | dietary | male | 6 | 3 | 6 | 7 | 6 | 7 | 6 | 7 | 6 | 5 | 6 | 5 | 2 | 5.54 |
| 12 | activity | male | 6 | 4 | 4 | 5 | 4 | 4 | 5 | 6 | 4 | 3 | 5 | 3 | 2 | 4.23 |
| 13 | dietary | male | 7 | 6 | 6 | 4 | 6 | 5 | 6 | 5 | 7 | 5 | 4 | 6 | 1 | 5.23 |
| 13 | activity | male | 7 | 6 | 6 | 7 | 7 | 7 | 7 | 7 | 6 | 7 | 7 | 7 | 1 | 6.31 |
| 14 | dietary | male | 4 | 6 | 6 | 7 | 1 | 2 | 3 | 7 | 6 | 0 | 1 | 2 | 6 | 3.92 |
| 14 | activity | male | 4 | 6 | 6 | 7 | 1 | 2 | 3 | 6 | 6 | 0 | 1 | 1 | 5 | 3.69 |
| 15 | dietary | male | 5 | 6 | 7 | 7 | 6 | 7 | 5 | 5 | 5 | 7 | 6 | 6 | 3 | 5.77 |
| 15 | activity | male | 4 | 6 | 6 | 7 | 3 | 6 | 5 | 6 | 6 | 3 | 3 | 6 | 3 | 4.92 |
| 16 | dietary | male | 6 | 5 | 7 | 6 | 6 | 7 | 7 | 7 | 6 | 5 | 6 | 3 | 0 | 5.46 |
| 16 | activity | male | 7 | 6 | 5 | 4 | 5 | 7 | 7 | 7 | 7 | 7 | 6 | 3 | 0 | 5.46 |
| 17 | dietary | male | 0 | 0 | 6 | 7 | 7 | 5 | 7 | 6 | 4 | 7 | 7 | 7 | 3 | 5.08 |
| 17 | activity | male | 0 | 0 | 2 | 4 | 4 | 2 | 4 | 4 | 3 | 2 | 1 | 3 | 1 | 2.31 |
| 18 | dietary | male | 4 | 5 | 7 | 5 | 7 | 7 | 6 | 6 | 7 | 7 | 7 | 6 | 3 | 5.92 |
| 18 | activity | male | 4 | 4 | 6 | 6 | 4 | 5 | 4 | 3 | 3 | 6 | 3 | 7 | 3 | 4.46 |
| 19 | dietary | male | 6 | 6 | 7 | 6 | 6 | 3 | 3 | 6 | 5 | 7 | 7 | 6 | 2 | 5.38 |
| 19 | activity | male | 7 | 7 | 7 | 7 | 5 | 7 | 6 | 6 | 6 | 3 | 4 | 4 | 0 | 5.31 |
| 20 | dietary | male | 6 | 6 | 5 | 6 | 4 | 7 | 6 | 5 | 6 | 5 | 7 | 4 | 3 | 5.38 |
| 20 | activity | male | 7 | 7 | 7 | 6 | 6 | 7 | 7 | 7 | 7 | 7 | 7 | 6 | 3 | 6.46 |
| 21 | dietary | male | 5 | 6 | 5 | 7 | 7 | 7 | 7 | 7 | 7 | 7 | 4 | 7 | 0 | 5.85 |
| 21 | activity | male | 7 | 7 | 7 | 4 | 6 | 7 | 7 | 7 | 7 | 6 | 5 | 5 | 0 | 5.77 |
| 22 | dietary | male | 6 | 6 | 3 | 3 | 7 | 6 | 6 | 7 | 7 | 7 | 6 | 7 | 0 | 5.46 |
| 22 | activity | male | 7 | 7 | 3 | 3 | 7 | 7 | 6 | 7 | 7 | 7 | 6 | 7 | 0 | 5.69 |
| 23 | dietary | male | 5 | 6 | 5 | 6 | 6 | 3 | 7 | 6 | 6 | 7 | 7 | 6 | 2 | 5.54 |
| 23 | activity | male | 6 | 5 | 4 | 6 | 6 | 2 | 0 | 4 | 4 | 5 | 3 | 5 | 1 | 3.92 |
| 24 | dietary | male | 5 | 6 | 6 | 5 | 5 | 4 | 7 | 6 | 7 | 6 | 6 | 6 | 0 | 5.31 |
| 24 | activity | male | 7 | 6 | 6 | 7 | 7 | 7 | 7 | 7 | 7 | 7 | 7 | 7 | 0 | 6.31 |
| 25 | dietary | male | 7 | 6 | 5 | 5 | 7 | 6 | 5 | 6 | 6 | 7 | 7 | 7 | 1 | 5.77 |
| 25 | activity | male | 1 | 1 | 2 | 4 | 2 | 4 | 2 | 3 | 7 | 7 | 7 | 7 | 1 | 3.69 |
| 26 | dietary | male | 7 | 6 | 6 | 5 | 5 | 4 | 7 | 5 | 5 | 5 | 3 | 0 | 0 | 4.46 |
| 26 | activity | male | 4 | 6 | 5 | 5 | 7 | 5 | 5 | 4 | 6 | 7 | 4 | 0 | 0 | 4.46 |
| 27 | dietary | male | 7 | 5 | 6 | 5 | 6 | 7 | 7 | 7 | 7 | 7 | 7 | 5 | 2 | 6 |
| 27 | activity | male | 7 | 7 | 6 | 6 | 5 | 5 | 6 | 7 | 7 | 7 | 7 | 4 | 2 | 5.85 |
| 28 | dietary | male | 1 | 0 | 0 | 2 | 0 | 0 | 0 | 0 | 0 | 0 | 0 | 0 | 0 | 0.23 |
| 28 | activity | male | 3 | 2 | 0 | 4 | 1 | 2 | 2 | 1 | 4 | 0 | 2 | 0 | 0 | 1.62 |
| 29 | dietary | male | 7 | 7 | 7 | 0 | 0 | 0 | 0 | 0 | 0 | 0 | 0 | 0 | 0 | 1.62 |
| 29 | activity | male | 7 | 7 | 7 | 0 | 0 | 1 | 1 | 1 | 2 | 3 | 0 | 2 | 0 | 2.38 |
| 30 | dietary | male | 3 | 6 | 6 | 7 | 7 | 6 | 6 | 7 | 1 | 6 | 5 | 0 | 0 | 4.62 |
| 30 | activity | male | 3 | 7 | 5 | 7 | 6 | 6 | 7 | 6 | 6 | 6 | 7 | 0 | 0 | 5.08 |
| 31 | dietary | male | 7 | 7 | 7 | 7 | 7 | 7 | 6 | 7 | 7 | 7 | 7 | 7 | 1 | 6.46 |
| 31 | activity | male | 6 | 6 | 7 | 4 | 7 | 7 | 7 | 7 | 7 | 5 | 7 | 7 | 1 | 6 |
| 32 | dietary | male | 3 | 2 | 7 | 5 | 6 | 6 | 6 | 6 | 4 | 5 | 6 | 7 | 6 | 5.31 |
| 32 | activity | male | 5 | 2 | 7 | 6 | 7 | 7 | 5 | 7 | 7 | 7 | 7 | 7 | 7 | 6.23 |
| 33 | dietary | male | 6 | 3 | 6 | 7 | 5 | 5 | 7 | 6 | 6 | 5 | 7 | 7 | 2 | 5.54 |
| 33 | activity | male | 6 | 5 | 7 | 7 | 7 | 7 | 6 | 7 | 7 | 7 | 7 | 7 | 2 | 6.31 |
| 34 | dietary | male | 7 | 5 | 7 | 6 | 6 | 7 | 6 | 7 | 5 | 6 | 6 | 5 | 2 | 5.77 |
| 34 | activity | male | 7 | 7 | 7 | 6 | 7 | 7 | 6 | 7 | 4 | 6 | 7 | 7 | 0 | 6 |
| 35 | dietary | male | 5 | 4 | 5 | 6 | 7 | 7 | 7 | 6 | 7 | 7 | 6 | 5 | 7 | 6.08 |
| 35 | activity | male | 7 | 6 | 6 | 5 | 6 | 7 | 6 | 4 | 5 | 7 | 7 | 6 | 6 | 6 |
| 36 | dietary | male | 7 | 6 | 7 | 6 | 4 | 6 | 4 | 0 | 0 | 0 | 0 | 0 | 0 | 3.08 |
| 36 | activity | male | 3 | 5 | 7 | 5 | 7 | 4 | 6 | 4 | 4 | 5 | 4 | 4 | 0 | 4.46 |
| 37 | dietary | male | 0 | 1 | 0 | 0 | 0 | 0 | 0 | 0 | 0 | 0 | 0 | 0 | 0 | 0.08 |
| 37 | activity | male | 1 | 2 | 2 | 0 | 0 | 3 | 2 | 0 | 0 | 0 | 0 | 0 | 0 | 0.77 |
| 38 | dietary | male | 4 | 3 | 5 | 4 | 4 | 6 | 4 | 3 | 4 | 3 | 6 | 3 | 3 | 4 |
| 38 | activity | male | 5 | 7 | 6 | 4 | 5 | 5 | 5 | 2 | 5 | 4 | 6 | 6 | 3 | 4.85 |
| 39 | dietary | male | 6 | 3 | 4 | 0 | 5 | 2 | 4 | 3 | 0 | 0 | 5 | 0 | 0 | 2.46 |
| 39 | activity | male | 7 | 4 | 4 | 0 | 5 | 0 | 7 | 7 | 0 | 0 | 6 | 0 | 0 | 3.08 |
| 40 | dietary | male | 5 | 5 | 6 | 5 | 6 | 6 | 6 | 5 | 6 | 6 | 6 | 6 | 3 | 5.46 |
| 40 | activity | male | 6 | 7 | 6 | 6 | 7 | 7 | 7 | 6 | 6 | 7 | 6 | 7 | 3 | 6.23 |
| 41 | dietary | male | 5 | 6 | 3 | 6 | 4 | 6 | 6 | 4 | 6 | 7 | 6 | 5 | 2 | 5.08 |
| 41 | activity | male | 6 | 6 | 6 | 6 | 6 | 7 | 7 | 7 | 7 | 6 | 6 | 7 | 2 | 6.08 |
| 42 | dietary | male | 6 | 6 | 5 | 6 | 4 | 5 | 6 | 7 | 7 | 5 | 5 | 6 | 5 | 5.62 |
| 42 | activity | male | 6 | 5 | 5 | 4 | 5 | 7 | 7 | 6 | 6 | 0 | 5 | 4 | 7 | 5.15 |
| 43 | dietary | male | 5 | 7 | 7 | 3 | 5 | 6 | 6 | 4 | 6 | 7 | 6 | 7 | 0 | 5.31 |
| 43 | activity | male | 6 | 7 | 7 | 4 | 7 | 7 | 7 | 5 | 7 | 7 | 4 | 6 | 0 | 5.69 |
| 44 | dietary | male | 6 | 5 | 7 | 7 | 5 | 7 | 7 | 6 | 6 | 5 | 4 | 6 | 7 | 6 |
| 44 | activity | male | 6 | 7 | 7 | 7 | 5 | 6 | 7 | 5 | 6 | 7 | 6 | 3 | 6 | 6 |
| 45 | dietary | male | 0 | 5 | 6 | 3 | 3 | 4 | 6 | 5 | 6 | 5 | 4 | 3 | 0 | 3.85 |
| 45 | activity | male | 0 | 7 | 6 | 3 | 4 | 5 | 7 | 6 | 6 | 4 | 3 | 2 | 0 | 4.08 |
| 46 | dietary | male | 6 | 5 | 6 | 5 | 4 | 5 | 6 | 5 | 6 | 5 | 4 | 3 | 0 | 4.62 |
| 46 | activity | male | 6 | 6 | 5 | 5 | 4 | 6 | 7 | 6 | 6 | 4 | 6 | 3 | 0 | 4.92 |
| 47 | dietary | male | 6 | 6 | 6 | 4 | 5 | 5 | 6 | 6 | 2 | 5 | 7 | 3 | 0 | 4.69 |
| 47 | activity | male | 6 | 6 | 5 | 5 | 6 | 6 | 6 | 4 | 2 | 4 | 7 | 3 | 0 | 4.62 |
| 48 | dietary | male | 5 | 6 | 7 | 6 | 6 | 6 | 7 | 7 | 7 | 6 | 7 | 6 | 2 | 6 |
| 48 | activity | male | 7 | 7 | 7 | 7 | 7 | 7 | 7 | 7 | 7 | 7 | 7 | 6 | 2 | 6.54 |
| 49 | dietary | male | 6 | 6 | 6 | 7 | 6 | 6 | 7 | 6 | 4 | 7 | 7 | 6 | 5 | 6.08 |
| 49 | activity | male | 6 | 6 | 7 | 7 | 7 | 7 | 6 | 7 | 7 | 7 | 7 | 7 | 6 | 6.69 |
| 50 | dietary | male | 7 | 7 | 6 | 6 | 7 | 7 | 5 | 6 | 6 | 6 | 7 | 7 | 2 | 6.08 |
| 50 | activity | male | 7 | 5 | 6 | 6 | 7 | 7 | 7 | 7 | 7 | 7 | 7 | 7 | 2 | 6.31 |
| 51 | dietary | male | 3 | 7 | 4 | 2 | 6 | 7 | 5 | 7 | 6 | 5 | 6 | 6 | 0 | 4.92 |
| 51 | activity | male | 6 | 7 | 7 | 7 | 6 | 7 | 6 | 7 | 6 | 7 | 6 | 7 | 0 | 6.08 |
| 52 | dietary | male | 7 | 7 | 7 | 6 | 7 | 6 | 7 | 7 | 7 | 7 | 7 | 7 | 0 | 6.31 |
| 52 | activity | male | 6 | 6 | 6 | 6 | 7 | 6 | 7 | 7 | 5 | 5 | 6 | 7 | 0 | 5.69 |
| 53 | dietary | male | 5 | 7 | 7 | 7 | 6 | 6 | 7 | 6 | 7 | 6 | 1 | 5 | 7 | 5.92 |
| 53 | activity | male | 7 | 6 | 5 | 6 | 6 | 4 | 5 | 6 | 7 | 5 | 1 | 6 | 5 | 5.31 |
| 54 | dietary | male | 6 | 5 | 4 | 4 | 5 | 6 | 7 | 6 | 5 | 7 | 1 | 7 | 4 | 5.15 |
| 54 | activity | male | 3 | 4 | 2 | 3 | 5 | 1 | 4 | 4 | 3 | 4 | 1 | 6 | 2 | 3.23 |
| 55 | dietary | male | 6 | 5 | 6 | 5 | 3 | 3 | 7 | 5 | 7 | 5 | 5 | 4 | 5 | 5.08 |
| 55 | activity | male | 6 | 7 | 7 | 7 | 7 | 5 | 7 | 7 | 7 | 7 | 7 | 5 | 7 | 6.62 |
| 56 | dietary | male | 4 | 7 | 7 | 6 | 6 | 3 | 3 | 6 | 4 | 7 | 7 | 7 | 0 | 5.15 |
| 56 | activity | male | 4 | 7 | 6 | 4 | 6 | 5 | 6 | 7 | 6 | 5 | 0 | 6 | 0 | 4.77 |
| 57 | dietary | male | 6 | 4 | 5 | 5 | 4 | 4 | 5 | 6 | 6 | 7 | 5 | 5 | 1 | 4.85 |
| 57 | activity | male | 7 | 6 | 7 | 6 | 5 | 5 | 7 | 5 | 7 | 5 | 6 | 6 | 1 | 5.62 |
| 58 | dietary | male | 3 | 5 | 4 | 6 | 4 | 7 | 5 | 3 | 3 | 3 | 3 | 6 | 0 | 4 |
| 58 | activity | male | 6 | 7 | 6 | 7 | 7 | 7 | 7 | 4 | 7 | 5 | 6 | 6 | 0 | 5.77 |
| 59 | dietary | male | 7 | 1 | 0 | 5 | 7 | 7 | 0 | 0 | 0 | 0 | 0 | 0 | 1 | 2.15 |
| 59 | activity | male | 7 | 1 | 0 | 5 | 7 | 7 | 7 | 7 | 5 | 2 | 4 | 1 | 0 | 4.08 |
| 60 | dietary | male | 4 | 3 | 5 | 5 | 4 | 4 | 3 | 4 | 4 | 4 | 4 | 4 | 2 | 3.85 |
| 60 | activity | male | 7 | 7 | 7 | 7 | 0 | 7 | 7 | 7 | 7 | 7 | 7 | 0 | 0 | 5.38 |
| 61 | dietary | male | 7 | 6 | 6 | 5 | 7 | 5 | 7 | 7 | 6 | 5 | 7 | 7 | 6 | 6.23 |
| 61 | activity | male | 5 | 5 | 6 | 7 | 6 | 5 | 6 | 6 | 4 | 5 | 7 | 7 | 5 | 5.69 |
| 62 | dietary | male | 5 | 5 | 5 | 5 | 6 | 6 | 3 | 7 | 5 | 5 | 6 | 6 | 1 | 5 |
| 62 | activity | male | 5 | 5 | 5 | 1 | 6 | 5 | 4 | 7 | 4 | 6 | 5 | 5 | 1 | 4.54 |
| 63 | dietary | male | 6 | 7 | 7 | 6 | 7 | 6 | 6 | 4 | 6 | 7 | 7 | 7 | 3 | 6.08 |
| 63 | activity | male | 7 | 5 | 5 | 6 | 6 | 6 | 7 | 6 | 7 | 7 | 5 | 6 | 3 | 5.85 |
| 64 | dietary | male | 7 | 7 | 6 | 5 | 5 | 6 | 7 | 7 | 6 | 6 | 6 | 3 | 0 | 5.46 |
| 64 | activity | male | 5 | 6 | 2 | 4 | 2 | 2 | 7 | 5 | 7 | 4 | 5 | 2 | 0 | 3.92 |
| 65 | dietary | male | 6 | 6 | 6 | 6 | 5 | 6 | 5 | 3 | 5 | 7 | 5 | 5 | 5 | 5.38 |
| 65 | activity | male | 7 | 5 | 5 | 5 | 3 | 6 | 6 | 7 | 5 | 5 | 6 | 7 | 7 | 5.69 |
| 66 | dietary | male | 2 | 5 | 6 | 7 | 3 | 4 | 6 | 7 | 6 | 6 | 7 | 7 | 5 | 5.46 |
| 66 | activity | male | 4 | 3 | 5 | 6 | 4 | 3 | 6 | 6 | 6 | 5 | 4 | 4 | 6 | 4.77 |
| 67 | dietary | male | 5 | 7 | 6 | 7 | 7 | 7 | 6 | 7 | 7 | 7 | 7 | 7 | 0 | 6.15 |
| 67 | activity | male | 6 | 6 | 3 | 6 | 6 | 7 | 6 | 6 | 7 | 7 | 7 | 7 | 0 | 5.69 |
| 68 | dietary | female | 5 | 4 | 4 | 4 | 5 | 6 | 4 | 5 | 4 | 5 | 4 | 5 | 0 | 4.23 |
| 68 | activity | female | 6 | 7 | 7 | 6 | 6 | 7 | 6 | 6 | 6 | 6 | 7 | 5 | 0 | 5.77 |
| 69 | dietary | female | 7 | 7 | 7 | 7 | 6 | 7 | 7 | 7 | 7 | 7 | 7 | 7 | 0 | 6.38 |
| 69 | activity | female | 4 | 6 | 5 | 6 | 6 | 5 | 5 | 6 | 4 | 5 | 7 | 7 | 0 | 5.08 |
| 70 | dietary | female | 6 | 6 | 6 | 7 | 7 | 4 | 6 | 5 | 7 | 7 | 5 | 7 | 1 | 5.69 |
| 70 | activity | female | 6 | 7 | 6 | 7 | 6 | 4 | 5 | 7 | 7 | 7 | 5 | 7 | 1 | 5.77 |
| 71 | dietary | female | 6 | 6 | 3 | 4 | 4 | 3 | 3 | 4 | 4 | 7 | 4 | 2 | 0 | 3.85 |
| 71 | activity | female | 4 | 6 | 3 | 3 | 4 | 5 | 5 | 6 | 3 | 3 | 1 | 2 | 0 | 3.46 |
| 72 | dietary | female | 6 | 6 | 7 | 6 | 7 | 4 | 3 | 5 | 4 | 7 | 7 | 7 | 3 | 5.54 |
| 72 | activity | female | 5 | 6 | 6 | 6 | 6 | 5 | 4 | 5 | 6 | 5 | 5 | 7 | 3 | 5.31 |
| 73 | dietary | female | 6 | 6 | 7 | 6 | 7 | 7 | 7 | 6 | 5 | 6 | 7 | 7 | 1 | 6 |
| 73 | activity | female | 6 | 7 | 7 | 5 | 7 | 6 | 7 | 7 | 7 | 7 | 7 | 7 | 1 | 6.23 |
| 74 | dietary | female | 0 | 0 | 0 | 0 | 0 | 0 | 0 | 0 | 0 | 0 | 0 | 0 | 0 | 0 |
| 74 | activity | female | 0 | 4 | 4 | 5 | 2 | 2 | 3 | 3 | 5 | 1 | 5 | 2 | 0 | 2.77 |
| 75 | dietary | female | 5 | 5 | 2 | 5 | 7 | 5 | 6 | 6 | 5 | 5 | 5 | 7 | 0 | 4.85 |
| 75 | activity | female | 3 | 4 | 3 | 7 | 7 | 7 | 7 | 7 | 7 | 7 | 7 | 6 | 0 | 5.54 |
| 76 | dietary | female | 7 | 5 | 7 | 7 | 6 | 7 | 4 | 4 | 3 | 4 | 2 | 6 | 6 | 5.23 |
| 76 | activity | female | 7 | 6 | 7 | 7 | 7 | 7 | 4 | 5 | 3 | 4 | 7 | 6 | 6 | 5.85 |
| 77 | dietary | female | 4 | 3 | 3 | 3 | 4 | 5 | 4 | 6 | 5 | 5 | 6 | 4 | 3 | 4.23 |
| 77 | activity | female | 7 | 7 | 7 | 7 | 7 | 7 | 7 | 7 | 7 | 7 | 7 | 7 | 3 | 6.69 |
| 78 | dietary | female | 4 | 4 | 2 | 4 | 3 | 4 | 2 | 2 | 2 | 2 | 0 | 0 | 0 | 2.23 |
| 78 | activity | female | 4 | 4 | 5 | 4 | 4 | 5 | 3 | 5 | 2 | 1 | 0 | 0 | 0 | 2.85 |
| 79 | dietary | female | 6 | 6 | 6 | 3 | 6 | 5 | 5 | 5 | 7 | 5 | 4 | 5 | 1 | 4.92 |
| 79 | activity | female | 7 | 6 | 6 | 7 | 7 | 7 | 7 | 7 | 7 | 7 | 6 | 7 | 1 | 6.31 |
| 80 | dietary | female | 6 | 6 | 6 | 6 | 7 | 5 | 4 | 7 | 6 | 6 | 7 | 5 | 6 | 5.92 |
| 80 | activity | female | 6 | 6 | 6 | 6 | 7 | 5 | 4 | 6 | 4 | 6 | 7 | 4 | 6 | 5.62 |
| 81 | dietary | female | 7 | 3 | 7 | 6 | 6 | 5 | 7 | 7 | 7 | 6 | 5 | 6 | 3 | 5.77 |
| 81 | activity | female | 7 | 7 | 7 | 7 | 7 | 7 | 7 | 7 | 7 | 7 | 7 | 7 | 3 | 6.69 |
| 82 | dietary | female | 0 | 0 | 0 | 0 | 0 | 0 | 0 | 0 | 0 | 0 | 0 | 0 | 0 | 0 |
| 82 | activity | female | 6 | 5 | 4 | 6 | 4 | 6 | 5 | 6 | 7 | 6 | 5 | 5 | 5 | 5.38 |
| 83 | dietary | female | 5 | 6 | 6 | 6 | 6 | 6 | 6 | 7 | 5 | 7 | 6 | 5 | 0 | 5.46 |
| 83 | activity | female | 5 | 5 | 4 | 6 | 4 | 5 | 4 | 3 | 4 | 3 | 4 | 4 | 0 | 3.92 |
| 84 | dietary | female | 5 | 6 | 7 | 6 | 7 | 7 | 6 | 7 | 7 | 7 | 7 | 7 | 3 | 6.31 |
| 84 | activity | female | 5 | 4 | 7 | 5 | 5 | 5 | 5 | 4 | 3 | 4 | 4 | 4 | 1 | 4.31 |
| 85 | dietary | female | 6 | 5 | 5 | 4 | 3 | 5 | 7 | 5 | 5 | 4 | 4 | 5 | 1 | 4.54 |
| 85 | activity | female | 7 | 7 | 6 | 6 | 6 | 5 | 5 | 4 | 5 | 7 | 7 | 1 | 3 | 5.31 |
| 86 | dietary | female | 7 | 7 | 6 | 7 | 7 | 7 | 7 | 6 | 2 | 7 | 5 | 0 | 0 | 5.23 |
| 86 | activity | female | 6 | 6 | 4 | 4 | 6 | 5 | 5 | 6 | 3 | 5 | 5 | 0 | 0 | 4.23 |
| 87 | dietary | female | 6 | 5 | 5 | 5 | 5 | 6 | 6 | 7 | 7 | 7 | 6 | 7 | 0 | 5.54 |
| 87 | activity | female | 7 | 7 | 7 | 7 | 7 | 7 | 7 | 7 | 7 | 7 | 7 | 5 | 0 | 6.31 |
| 88 | dietary | female | 6 | 6 | 3 | 3 | 7 | 6 | 7 | 7 | 7 | 7 | 6 | 7 | 0 | 5.54 |
| 88 | activity | female | 7 | 7 | 3 | 3 | 7 | 7 | 7 | 7 | 7 | 7 | 6 | 7 | 0 | 5.77 |
| 89 | dietary | female | 5 | 5 | 3 | 7 | 7 | 7 | 7 | 5 | 4 | 6 | 6 | 4 | 3 | 5.31 |
| 89 | activity | female | 4 | 5 | 3 | 3 | 7 | 0 | 1 | 0 | 6 | 3 | 4 | 5 | 3 | 3.38 |
| 90 | dietary | female | 7 | 6 | 7 | 3 | 7 | 6 | 7 | 6 | 4 | 6 | 7 | 4 | 3 | 5.62 |
| 90 | activity | female | 5 | 6 | 6 | 6 | 6 | 6 | 7 | 7 | 7 | 6 | 6 | 6 | 2 | 5.85 |
| 91 | dietary | female | 7 | 6 | 7 | 5 | 6 | 6 | 7 | 5 | 7 | 7 | 7 | 6 | 1 | 5.92 |
| 91 | activity | female | 2 | 1 | 3 | 6 | 5 | 6 | 4 | 3 | 6 | 6 | 5 | 6 | 1 | 4.15 |
| 92 | dietary | female | 0 | 0 | 0 | 0 | 0 | 0 | 0 | 0 | 0 | 0 | 0 | 0 | 0 | 0 |
| 92 | activity | female | 6 | 6 | 6 | 5 | 6 | 5 | 7 | 4 | 7 | 7 | 6 | 7 | 7 | 6.08 |
| 93 | dietary | female | 6 | 5 | 6 | 5 | 7 | 6 | 7 | 7 | 2 | 5 | 3 | 0 | 0 | 4.54 |
| 93 | activity | female | 7 | 7 | 5 | 6 | 7 | 6 | 5 | 4 | 7 | 6 | 4 | 0 | 0 | 4.92 |
| 94 | dietary | female | 6 | 3 | 7 | 4 | 7 | 6 | 5 | 6 | 5 | 4 | 6 | 6 | 6 | 5.46 |
| 94 | activity | female | 5 | 4 | 6 | 7 | 5 | 4 | 7 | 6 | 7 | 7 | 5 | 7 | 3 | 5.62 |
| 95 | dietary | female | 2 | 3 | 2 | 1 | 1 | 2 | 0 | 0 | 0 | 3 | 0 | 2 | 0 | 1.23 |
| 95 | activity | female | 2 | 1 | 2 | 2 | 0 | 0 | 0 | 0 | 1 | 2 | 0 | 2 | 0 | 0.92 |
| 96 | dietary | female | 5 | 5 | 4 | 6 | 5 | 4 | 2 | 4 | 5 | 6 | 6 | 6 | 1 | 4.54 |
| 96 | activity | female | 6 | 6 | 5 | 4 | 5 | 6 | 3 | 4 | 7 | 6 | 7 | 7 | 1 | 5.15 |
| 97 | dietary | female | 7 | 6 | 7 | 7 | 7 | 7 | 7 | 7 | 7 | 7 | 7 | 7 | 1 | 6.46 |
| 97 | activity | female | 6 | 7 | 7 | 6 | 7 | 7 | 7 | 7 | 7 | 7 | 7 | 7 | 1 | 6.38 |
| 98 | dietary | female | 5 | 7 | 6 | 6 | 6 | 6 | 6 | 7 | 7 | 6 | 6 | 7 | 6 | 6.23 |
| 98 | activity | female | 5 | 7 | 7 | 7 | 7 | 7 | 7 | 7 | 7 | 7 | 7 | 1 | 7 | 6.38 |
| 99 | dietary | female | 0 | 0 | 0 | 0 | 0 | 0 | 0 | 0 | 0 | 0 | 0 | 0 | 0 | 0 |
| 99 | activity | female | 4 | 4 | 5 | 5 | 5 | 4 | 4 | 5 | 5 | 4 | 3 | 5 | 0 | 4.08 |
| 100 | dietary | female | 7 | 5 | 7 | 6 | 7 | 7 | 7 | 7 | 6 | 6 | 5 | 5 | 7 | 6.31 |
| 100 | activity | female | 7 | 7 | 7 | 6 | 7 | 7 | 7 | 7 | 7 | 6 | 7 | 7 | 7 | 6.85 |
| 101 | dietary | female | 6 | 6 | 7 | 7 | 7 | 7 | 7 | 7 | 7 | 7 | 7 | 6 | 7 | 6.77 |
| 101 | activity | female | 7 | 7 | 5 | 4 | 6 | 5 | 6 | 5 | 4 | 5 | 5 | 5 | 5 | 5.31 |
| 102 | dietary | female | 6 | 7 | 6 | 6 | 7 | 6 | 6 | 5 | 4 | 7 | 7 | 7 | 3 | 5.92 |
| 102 | activity | female | 5 | 7 | 6 | 6 | 4 | 7 | 7 | 4 | 5 | 7 | 6 | 7 | 3 | 5.69 |
| 103 | dietary | female | 4 | 3 | 3 | 4 | 3 | 5 | 4 | 2 | 2 | 4 | 2 | 3 | 0 | 3 |
| 103 | activity | female | 5 | 7 | 7 | 7 | 5 | 6 | 5 | 4 | 7 | 5 | 6 | 3 | 0 | 5.15 |
| 104 | dietary | female | 6 | 4 | 4 | 5 | 4 | 3 | 5 | 4 | 5 | 5 | 5 | 7 | 1 | 4.46 |
| 104 | activity | female | 7 | 7 | 7 | 6 | 7 | 7 | 7 | 5 | 6 | 6 | 7 | 6 | 1 | 6.08 |
| 105 | dietary | female | 3 | 4 | 2 | 5 | 4 | 4 | 5 | 4 | 6 | 6 | 5 | 7 | 3 | 4.46 |
| 105 | activity | female | 5 | 5 | 6 | 5 | 6 | 6 | 7 | 7 | 6 | 4 | 5 | 5 | 2 | 5.31 |
| 106 | dietary | female | 6 | 6 | 5 | 6 | 4 | 5 | 5 | 7 | 7 | 7 | 6 | 6 | 5 | 5.77 |
| 106 | activity | female | 7 | 6 | 5 | 5 | 6 | 7 | 6 | 7 | 7 | 7 | 5 | 5 | 7 | 6.15 |
| 107 | dietary | female | 5 | 5 | 4 | 7 | 5 | 5 | 4 | 3 | 5 | 2 | 1 | 4 | 1 | 3.92 |
| 107 | activity | female | 7 | 6 | 7 | 7 | 5 | 5 | 4 | 3 | 7 | 5 | 4 | 5 | 4 | 5.31 |
| 108 | dietary | female | 4 | 7 | 7 | 7 | 6 | 5 | 6 | 7 | 7 | 5 | 4 | 6 | 7 | 6 |
| 108 | activity | female | 5 | 6 | 7 | 6 | 7 | 5 | 5 | 7 | 7 | 6 | 5 | 4 | 4 | 5.69 |
| 109 | dietary | female | 5 | 6 | 7 | 6 | 3 | 4 | 7 | 7 | 5 | 7 | 6 | 7 | 7 | 5.92 |
| 109 | activity | female | 5 | 5 | 4 | 7 | 4 | 6 | 6 | 6 | 6 | 3 | 1 | 1 | 6 | 4.62 |
| 110 | dietary | female | 6 | 5 | 6 | 5 | 6 | 6 | 6 | 6 | 6 | 5 | 5 | 3 | 0 | 5 |
| 110 | activity | female | 0 | 0 | 2 | 6 | 4 | 6 | 6 | 6 | 6 | 4 | 6 | 3 | 0 | 3.77 |
| 111 | dietary | female | 6 | 7 | 6 | 7 | 7 | 7 | 7 | 7 | 7 | 7 | 7 | 7 | 0 | 6.31 |
| 111 | activity | female | 6 | 7 | 7 | 6 | 7 | 7 | 7 | 7 | 7 | 7 | 7 | 7 | 0 | 6.31 |
| 112 | dietary | female | 7 | 7 | 7 | 6 | 7 | 7 | 5 | 7 | 7 | 6 | 0 | 6 | 6 | 6 |
| 112 | activity | female | 6 | 7 | 7 | 7 | 7 | 7 | 7 | 7 | 7 | 7 | 0 | 7 | 7 | 6.38 |
| 113 | dietary | female | 7 | 7 | 5 | 6 | 0 | 7 | 4 | 6 | 0 | 1 | 0 | 0 | 0 | 3.31 |
| 113 | activity | female | 7 | 7 | 4 | 4 | 0 | 6 | 2 | 7 | 0 | 1 | 7 | 6 | 0 | 3.92 |
| 114 | dietary | female | 5 | 7 | 6 | 5 | 6 | 7 | 6 | 2 | 7 | 7 | 7 | 4 | 6 | 5.77 |
| 114 | activity | female | 7 | 7 | 5 | 5 | 5 | 7 | 6 | 7 | 7 | 7 | 7 | 7 | 5 | 6.31 |
| 115 | dietary | female | 5 | 4 | 4 | 7 | 3 | 4 | 6 | 6 | 4 | 5 | 5 | 5 | 2 | 4.62 |
| 115 | activity | female | 6 | 7 | 5 | 7 | 5 | 5 | 6 | 5 | 3 | 6 | 7 | 4 | 2 | 5.23 |
| 116 | dietary | female | 6 | 5 | 7 | 5 | 4 | 4 | 5 | 6 | 5 | 5 | 4 | 1 | 3 | 4.62 |
| 116 | activity | female | 5 | 7 | 2 | 4 | 2 | 2 | 5 | 5 | 0 | 0 | 5 | 1 | 2 | 3.08 |
| 117 | dietary | female | 5 | 4 | 5 | 5 | 4 | 6 | 5 | 5 | 7 | 6 | 5 | 6 | 2 | 5 |
| 117 | activity | female | 4 | 2 | 5 | 5 | 4 | 4 | 4 | 5 | 2 | 5 | 3 | 5 | 1 | 3.77 |
| 118 | dietary | female | 5 | 5 | 6 | 7 | 5 | 5 | 7 | 6 | 7 | 6 | 5 | 6 | 0 | 5.38 |
| 118 | activity | female | 6 | 7 | 6 | 6 | 5 | 4 | 5 | 5 | 6 | 5 | 5 | 6 | 0 | 5.08 |
| 119 | dietary | female | 7 | 7 | 7 | 6 | 7 | 7 | 7 | 7 | 7 | 7 | 6 | 7 | 7 | 6.85 |
| 119 | activity | female | 7 | 6 | 5 | 1 | 4 | 6 | 4 | 7 | 5 | 7 | 6 | 7 | 5 | 5.38 |
| 120 | dietary | female | 4 | 3 | 5 | 5 | 1 | 4 | 5 | 3 | 6 | 6 | 5 | 0 | 0 | 3.62 |
| 120 | activity | female | 7 | 5 | 7 | 5 | 4 | 5 | 5 | 7 | 7 | 6 | 7 | 0 | 0 | 5 |
| 121 | dietary | female | 0 | 0 | 0 | 0 | 0 | 0 | 0 | 0 | 0 | 0 | 0 | 0 | 0 | 0 |
| 121 | activity | female | 4 | 5 | 3 | 4 | 5 | 5 | 3 | 5 | 3 | 3 | 5 | 2 | 0 | 3.62 |
| 122 | dietary | female | 7 | 2 | 7 | 6 | 5 | 5 | 6 | 7 | 2 | 7 | 5 | 2 | 0 | 4.69 |
| 122 | activity | female | 4 | 6 | 4 | 7 | 7 | 5 | 7 | 3 | 6 | 6 | 7 | 2 | 0 | 4.92 |
| 123 | dietary | female | 2 | 4 | 5 | 7 | 2 | 0 | 5 | 7 | 7 | 4 | 7 | 7 | 2 | 4.54 |
| 123 | activity | female | 7 | 2 | 3 | 7 | 4 | 7 | 7 | 3 | 6 | 5 | 6 | 7 | 2 | 5.08 |
| 124 | dietary | female | 5 | 5 | 7 | 4 | 5 | 6 | 4 | 5 | 7 | 6 | 4 | 6 | 3 | 5.15 |
| 124 | activity | female | 5 | 7 | 7 | 5 | 5 | 6 | 5 | 5 | 5 | 7 | 7 | 7 | 2 | 5.62 |
| 125 | dietary | female | 5 | 5 | 5 | 4 | 4 | 6 | 6 | 4 | 5 | 6 | 1 | 5 | 1 | 4.38 |
| 125 | activity | female | 6 | 6 | 5 | 7 | 6 | 7 | 6 | 6 | 6 | 6 | 3 | 6 | 1 | 5.46 |
| 126 | dietary | female | 1 | 4 | 6 | 5 | 6 | 5 | 6 | 7 | 6 | 7 | 1 | 0 | 0 | 4.15 |
| 126 | activity | female | 2 | 5 | 7 | 7 | 7 | 7 | 7 | 7 | 7 | 7 | 1 | 0 | 0 | 4.92 |
| 127 | dietary | female | 6 | 6 | 6 | 6 | 5 | 7 | 7 | 7 | 4 | 5 | 6 | 5 | 1 | 5.46 |
| 127 | activity | female | 6 | 5 | 6 | 3 | 6 | 2 | 5 | 6 | 6 | 5 | 7 | 7 | 1 | 5 |
| 128 | dietary | female | 6 | 6 | 7 | 4 | 5 | 7 | 6 | 7 | 7 | 7 | 7 | 4 | 0 | 5.62 |
| 128 | activity | female | 5 | 5 | 5 | 5 | 6 | 6 | 7 | 4 | 2 | 1 | 6 | 4 | 0 | 4.31 |
| 129 | dietary | female | 6 | 6 | 5 | 6 | 6 | 5 | 6 | 5 | 4 | 5 | 6 | 7 | 5 | 5.54 |
| 129 | activity | female | 7 | 7 | 7 | 7 | 7 | 7 | 5 | 7 | 7 | 7 | 7 | 7 | 7 | 6.85 |
| 130 | dietary | male | NA | NA | NA | NA | NA | NA | NA | NA | NA | NA | NA | NA | NA | NA |
| 130 | activity | male | NA | NA | NA | NA | NA | NA | NA | NA | NA | NA | NA | NA | NA | NA |
| 131 | dietary | male | NA | NA | NA | NA | NA | NA | NA | NA | NA | NA | NA | NA | NA | NA |
| 131 | activity | male | NA | NA | NA | NA | NA | NA | NA | NA | NA | NA | NA | NA | NA | NA |
| 132 | dietary | female | NA | NA | NA | NA | NA | NA | NA | NA | NA | NA | NA | NA | NA | NA |
| 132 | activity | female | NA | NA | NA | NA | NA | NA | NA | NA | NA | NA | NA | NA | NA | NA |
| 133 | dietary | female | NA | NA | NA | NA | NA | NA | NA | NA | NA | NA | NA | NA | NA | NA |
| 133 | activity | female | NA | NA | NA | NA | NA | NA | NA | NA | NA | NA | NA | NA | NA | NA |
| 134 | dietary | female | NA | NA | NA | NA | NA | NA | NA | NA | NA | NA | NA | NA | NA | NA |
| 134 | activity | female | NA | NA | NA | NA | NA | NA | NA | NA | NA | NA | NA | NA | NA | NA |

# Supplementary Figures

Supplementary Figure 1: Flowchart of the participants screening and selection for the Growing Old TOgether study.

Supplementary Figure 2: Association of baseline lumbar spine BMD and baseline health marker scores. On the x-axis the z-scaled baseline metabolic health score is plotted. On the baseline lumbar spine BMD is plotted. The line through the data represents the estimated effect between the lumbar spine BMD change and the baseline metabolic health score, the grey ribbon represents the 95% confidence interval. The formula of the estimated effect and the significance level are plotted in the top-left corner of each panel.

Supplementary Figure 3: Fasting Vitamin D levels at baseline and post intervention, plotted per month of intervention starting date. X-axis represents the baseline fasting vitamin D levels. Y-axis represents the post intervention fasting vitamin D levels. Blue squares represent male samples, red circles represent female samples. Months indicate the starting month of the intervention.
